# Demethylation of methionine and keratin damage in human hair

**DOI:** 10.1007/s00726-018-2545-3

**Published:** 2018-02-26

**Authors:** Kamila Borowczyk, Joanna Suliburska, Hieronim Jakubowski

**Affiliations:** 1 Department of Microbiology, Biochemistry and Molecular Genetics, Rutgers-New Jersey Medical School, International Center for Public Health, 225 Warren Street, Newark, NJ 07103 USA; 2 Department of Environmental Chemistry, University of Łódź, 90-236 Łódź, Poland; 3 Department of Human Nutrition and Hygiene, Poznań University of Life Sciences, 60-632 Poznań, Poland; 4 Department of Biochemistry and Biotechnology, Poznań University of Life Sciences, 60-632 Poznań, Poland

**Keywords:** Hair keratin damage, Homocysteine, Methionine demethylation, Copper, Iron

## Abstract

Growing human head hair contains a history of keratin and provides a unique model for studies of protein damage. Here, we examined mechanism of homocysteine (Hcy) accumulation and keratin damage in human hair. We found that the content of Hcy-keratin increased along the hair fiber, with levels 5–10-fold higher levels in older sections at the hair’s tip than in younger sections at hair’s base. The accumulation of Hcy led to a complete loss of keratin solubility in sodium dodecyl sulfate. The increase in Hcy-keratin was accompanied by a decrease in methionine-keratin. Levels of Hcy-keratin were correlated with hair copper and iron in older hair. These relationships were recapitulated in model experiments in vitro, in which Hcy generation from Met exhibited a similar dependence on copper or iron. Taken together, these findings suggest that Hcy-keratin accumulation is due to copper/iron-catalyzed demethylation of methionine residues and contributes to keratin damage in human hair.

## Introduction

Homocysteine (Hcy) is a universal intermediate in the metabolism of two major sulfur-containing amino acids methionine (Met) and cysteine. While Met and cysteine (Cys) are canonical coded amino acids that are incorporated into proteins by the ribosomal biosynthetic apparatus, Hcy is not (Jakubowski 2017). However, although Hcy is a non‐coded amino acid, proteins carry Hcy residues linked via an isopeptide bond to lysine (Lys) residues (*N*-Hcy-protein) (Jakubowski 1999, 2002; Sikora et al. 2014) or via a disulfide bond to cysteine residues (*S*-Hcy-protein) (Lim et al. 2003; Jacovina et al. 2009). Proteins can also carry Hcy bound by a peptide bond than can be formed translationally by a nitric oxide-dependent mechanism (Jakubowski 2000a, b, 2001, 2017) or post-translationally by metal-dependent demethylation of a protein methionine residue (Mozziconacci et al. 2013). However, although about a dozen individual proteins that contain Hcy N-linked to lysine residues or S-linked to cysteine residues have been identified in vivo in humans or animals (Jakubowski 2013), proteins that carry Hcy bound by a peptide bond have not yet been identified.

Human head hair provides a unique model to address mechanisms of protein aging and damage. Because hair fibers grow at a relatively uniform rate of 1 cm/month (Nissimov et al. 2007), long hair provide a history of keratin from about a month at their base to several years at their tip. Long human hair is known to undergo damage, e.g. generating unsightly splitting ends, but the underlying mechanism is not fully understood. In the present work we have examined a hypothesis that the damage that accumulates in growing human hair is caused by the demethylation of keratin Met residues to Hcy.

## Materials and methods

### Reagents

l-Hcy-thiolactone·HCl, d,l-Hcy, l-methionine, *N*-acetyl-l-cysteine (NAC), horse spleen ferritin, dithiothreitol (DTT), tris-(2-carboxyethyl)phosphine (TCEP), *o*-phthaldialdehyde (OPA), monosodium phosphate, NaOH, sodium citrate, NaCl, CuCl_2_, FeCl_2_, FeCl_3_, vitamin C, sodium dodecyl sulfate (SDS), chloroform, methanol, HCl, trichloroacetic acid, were purchased from Sigma-Aldrich. Suprapure nitric acid, 65% and perchloric acid, 60% were from Merck. Reagents were prepared in MiliQ purified water.

### Human hair

Unaltered head hair was obtained from healthy individuals (females, *n* = 51, 0.5—84 years old; males, *n* = 30, 1.25—68 years old) recruited from the Poznań population. Shed hair was collected during regular daily combing. Their base was identified by the presence of the hair bulb and only those hairs with the bulb were used in experiments. A 1- or 3-cm segment of the hair shaft (~ 2 mg hair from 15 to 30 individual hairs) was used to quantify Hcy content. The age of a hair segment, estimated from its distance from the scalp, assuming an average hair growth rate of 1 cm/month (Nissimov et al. 2007), is indicated in figures. The terms “young hair” and “old hair” or “aged hair” define segments of long hair at their base (close to the scalp) and at the tip, respectively.

### Sample preparation for hair *S*-Hcy-keratin and Hcy-keratin assays

Samples were prepared by a modification of previously described procedures as described below. The inter-assay and intra-assay variabilities for the quantification of various forms of Hcy were 7.3 and 11.5% (Jakubowski 2002, 2008, 2016).

### *S*-Hcy-keratin

To liberate Hcy from *S*-Hcy-keratin, hair (~ 2 mg) were treated in a 0.5 mL Eppendorf polyethylene tube with a hot solution containing 50 mM Na_2_HPO_4_, 20 mM NaOH, 25 mM DTT, 1% SDS (200 μL, 65 °C, 1 h). The extract was collected by centrifugation, hair were similarly extracted with a fresh solution the second time, and the extracts combined (400 μL). Control experiments show that this procedure liberates > 95% Hcy present in hair *S*-Hcy-keratin. The extracts (40 μL) were treated with DTT (4 μL, 0.25 M) and HCl (4 μL, 12 N) on a heat block at 100 °C for 30 min to convert the liberated Hcy to Hcy-thiolactone, which was then quantified by HPLC (Jakubowski 2016). Authentic Hcy was similarly processed as a standard for *S*-Hcy assays.

Hair pellets after extraction were saved for SDS-insoluble *N*-Hcy-keratin quantification.

### SDS-soluble Hcy-keratin

Extracts (300 μL) of SDS-soluble Hcy-keratin were supplemented with 34 μL 100% trichloroacetic acid to precipitate keratin. Protein precipitates were collected by centrifugation, transferred to 1-mL Wheaton Gold Band ampoules, and hydrolyzed in 6 N HCl, 50 mM DTT (110 μL, 120 °C, 1 h). The hydrolysates were lyophilized on Labconco CentriVap (40 min, 70 °C), dissolved in 10 μL water, purified by two-dimensional TLC, and analyzed by HPLC.

### SDS-insoluble Hcy-keratin

Hair pellets containing SDS-insoluble Hcy-keratin were transferred to 1-mL Wheaton Gold Band ampoules and hydrolyzed in 6 N HCl, 50 mM DTT (110 μL, 120 °C, 1 h). The hydrolysates were lyophilized on a Labconco CentriVap (40 min, 70 °C), dissolved in 10 μL water, purified by two-dimensional TLC, and analyzed by HPLC.

### Total Hcy-keratin

The term “total Hcy-keratin’ refers to the content of all keratin-bound Hcy that is solubilized by acid hydrolysis (i.e., Hcy-keratin + *S*-Hcy-keratin). Hair (~ 2 mg) were hydrolyzed in 1-mL Wheaton Gold Band ampoules containing 6 N HCl, 50 mM DTT (100 μL, 120 °C, 1 h). The hydrolysates were lyophilized using a Labconco CentriVap, dissolved in 100 μL 0.5 M K_2_HPO_4_ on ice, and Hcy-thiolactone was purified by two-dimensional TLC. Alternatively, in some experiments Hcy-thiolactone was purified by a 5-min extraction of ice-cold chloroform/methanol (2:1, v/v, 400 μL). Organic layer containing Hcy-thiolactone (bottom), separated by a 1-min microcentrifugation at 4 °C, was collected and re-extracted with 150 μL 0.1 N HCl. Aqueous layer containing Hcy-thiolactone (top) was dried on a Labconco CentriVap (40 min, 70 °C), dissolved in 100 μL deionized water, and analyzed by HPLC.

Horse spleen ferritin, containing 0.49 mol *N*-Hcy/mol protein, was processed in parallel as a standard for *N*-Hcy (Jakubowski 2008, 2016). These procedures quantitatively liberate Hcy from *N*-Hcy-protein and convert the liberated Hcy to Hcy-thiolactone, which is then quantified by HPLC (Jakubowski 2002, 2008, 2016).

### HPLC, detection, and quantification

Quantification of Hcy-thiolactone generated from *N*-Hcy-keratin and/or *S*-Hcy-keratin was carried out by as previously described (Chwatko and Jakubowski 2005; Jakubowski 2008, 2016). Briefly, a Beckman-Coulter System Gold Nouveau HPLC instrumentation with a manual injector (7725i Rheodyne, with 0.1 mL loop) and a Jasco 1520 fluorescence detector were used. Chromatograms were analyzed using a Gold Nouveau chromatography workstation software for Windows. Samples were injected onto a cation-exchange poly-sulfoethyl aspartamide column (35 × 2 mm, 5 μm, 300 Å) (PolyLC, Inc.), eluted isocratically with 30 mM NaCl, 10 mM sodium phosphate buffer (pH 6.6) at a flow rate 0.6 mL/min. The effluent was mixed in a three-way tee with 2.5 mM OPA in 0.25 M NaOH, delivered at a flow rate 0.3 mL/min, the mixture passed through a reaction coil (Teflon tubing, 0.3 mm I.D. × 3 m), and the fluorescence at 480 nm was recorded (excitation 370 nm). Hcy-thiolactone eluted at 3 min and each run was completed in 4 min.

### Met-keratin and Hcy-keratin assays

Hair (~ 10 mg) were hydrolyzed in 1-mL Wheaton Gold Band ampoules containing 6 N HCl (100 μL, 120 °C, 1 h). The hydrolysates were dried out, dissolved in 50 μL 0.2 M sodium phosphate buffer (pH 7.4), reduced with 2 μL 0.25 M TCEP for 10 min, and supplemented with 10 μL 0.5 M NAC. Met and Hcy liberated from hair keratin were quantified by HPLC as previously described (Borowczyk et al. 2016). Briefly, a Hewlett-Packard (Waldbronn, Germany) 1100 Series system, controlled by HP ChemStation software, containing quaternary pump, auto-sampler, temperature control, vacuum degasser, and 1260 Series FL detector was used. Samples were injected on a reversed-phase PRP-1 column (150 × 4.6 mm, 5 μm; Hamilton, Energy Way, Reno, NV, USA) eluted at a flow rate 1 mL/min, 25 °C, with 0.01 M OPA, 0.1 M L − 1 NaOH (A) and acetonitrile (B) as follows: 0–8 min, 14–25% (B); 8–12 min, 25% (B), 12–14 min, 25–14% (B). Met and Hcy were identified by co-elution with the authentic standards, monitored by fluorescence from 0 to 7.2 min, exc. 348 nm, em. 438 nm for Met, and from 7.2 to 14 min, ex. 370 nm, em. 480 nm for Hcy. Elution times for Met and Hcy were 6.2 and 9.8 min, respectively.

### Iron and copper assays

Hair samples were mineralized with 65% nitric acid (Merck) in a microwave oven (Mars 5 Digestion Microwave System, CEM Corporation). Iron and copper were quantified by flame atomic absorption spectrometry using a Zeiss AAS-3 spectrometer with deuterium background correction as previously described (Suliburska 2011). The accuracy of the assay was 94% for iron and 102% for copper as verified by certified reference materials (Human Hair NCS DC73347a, LGC Standards).

### Demethylation of free Met to Hcy

Model reactions were carried out at 37 °C in Na-citrate buffer, pH 6.0, containing Met, copper or iron chloride salts, and vitamin C in a final volume of 400 μL under conditions specified in figure legends. Hcy generated from methionine was assayed by the conversion to Hcy-thiolactone, which was quantified by HPLC as previously described (Jakubowski 2016). Briefly, 5 μL aliquots of reaction mixtures were treated with 5 μL DTT, 10 μL water, and 10 μL 3 N HCl at 100 °C for 20 min. The assay mixtures were dried on a Labconco CentriVap, dissolved in 100 μL water, and analyzed by cation exchange HPLC. Authentic Hcy samples were similarly processed as standards. The inter-assay and intra-assay variabilities were 4.7 and 6.8% for Hcy, and 1.0 and 3.4% for Met, respectively.

### Statistical analysis

The results are reported as mean ± standard deviation. Comparisons between two groups are analyzed by using two-sided Student’s test. Relationships between Hcy-keratin and hair age or metal content were fitted to exponential equations and analyzed by linear regression. The level of statistical significance was set to *P* < 0.05. Analyses were carried out by using Sigma Plot software.

## Results

### Hair contain Hcy bound by amide/peptide and disulfide bonds

**Fig. 1:**
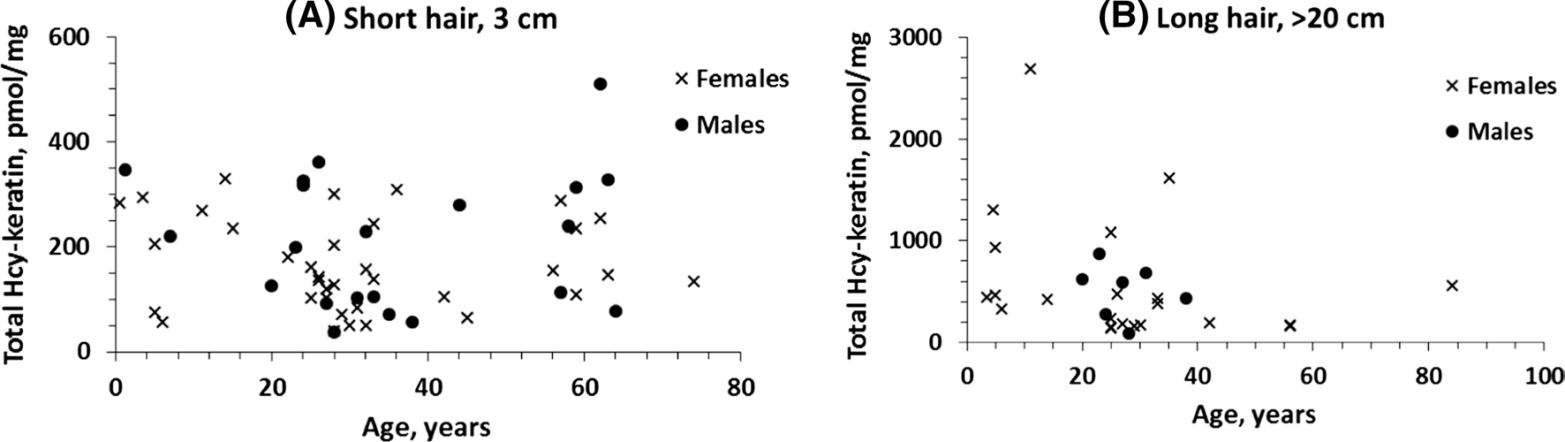
Relationships between human hair Hcy-keratin and the donor’s age. **a** Short hair (3 cm) and **b** long hair (> 20 cm)

To quantify Hcy, human hair samples (*n* = 76) were acid-hydrolyzed to liberate any Hcy that might be bound to keratin by amide/peptide or disulfide bonds. We found that each hair sample contained Hcy, which varied 14-fold between individuals (from 37 to 510 pmol/mg) and did not depend on individual’s age or gender (Fig. 1a). Mean ± SD Hcy values were similar for females and males, 176 ± 85 (*n* = 38) and 228 ± 133 pmol/mg (*n* = 21), respectively (Table 1).

**Table 1:** Total Hcy-keratin content increases at tips of long human hair

Gender (*n*)	Mean total Hcy-keratin ± SD, pmol/mg hair		
	Short hair, 3 cm (*n*)	Long hair, > 20 cm (*n*)	*P* value, long vs. short hair
Female	176 ± 85 (*n* = 38)	542 ± 615 (*n* = 21)	0.002
Male	228 ± 133 (*n* = 21)	508 ± 288 (7)	0.010

Free Hcy is unlikely to occur in human hair because it is frequently washed or otherwise exposed to water. Indeed, control experiments showed that of the 322 ± 134 pmol Hcy/mg present in samples of human hair (*n* = 4), only about 1% (4.4 ± 1.2 pmol Hcy/mg) was solubilized by the treatment with 20 mM K_2_HPO_4_. Because keratin is the major component of hair, accounting for 95% by weight (Robbins 2012), these findings suggest that Hcy is a natural component of human hair keratin.

### Total Hcy-keratin content increases along the hair fiber

Hair fibers grow at a relatively uniform rate of 1 cm/month. Thus, long hairs provide an aging history of keratin from about a month at hair’s base to several years at its tip. To determine how Hcy dynamics change during the hair growth, we quantified total Hcy-keratin content in short and long hair. We found that total Hcy-keratin was significantly elevated at the tips of long, i.e. old, hair fibers relative to the short, i.e. young, hair fibers. However, total Hcy-keratin level exhibited inter-individual variability that was independent of individual’s age (Fig. 1b) and gender (Table 1).

**Fig. 2:**
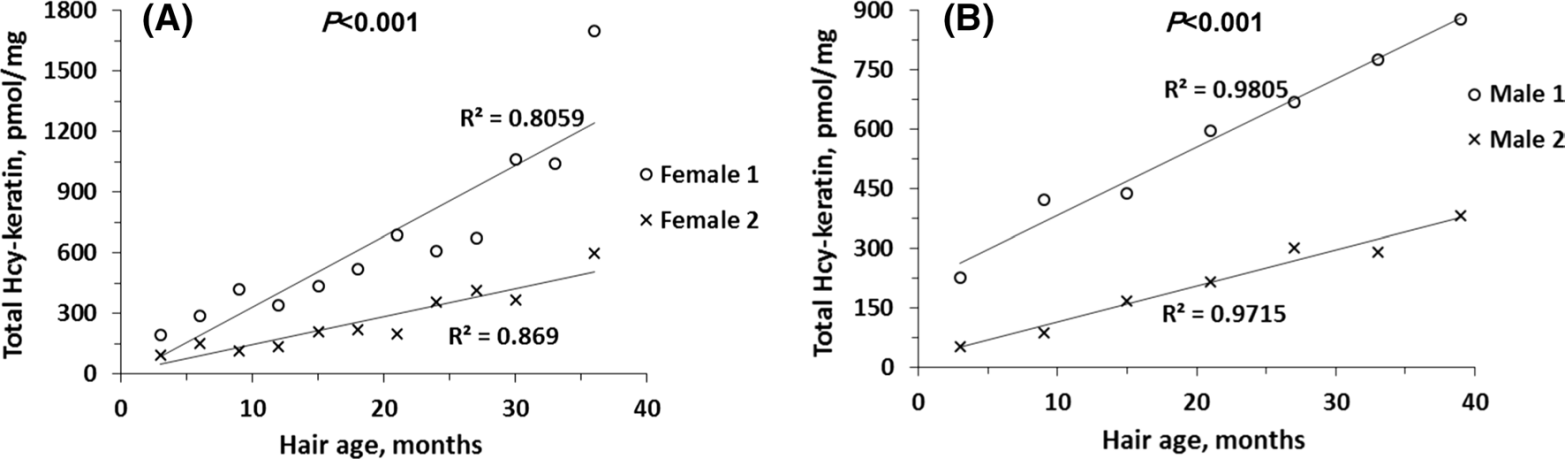
Relationships between hair total Hcy-keratin levels and hair age in individual human subjects. **a** Females; **b** males

We then quantified total Hcy-keratin content along human hair fibers as long as 50 cm, corresponding to hair’s age of 50 months at the tip. We found that in long hair total Hcy-keratin increased continuously along hair fibers, reaching 5–10-fold higher levels at their tips (i.e. in old hair), compared with the levels at their base close to the scalp (i.e. in young hair), both in females (Fig. 2a) and males (Fig. 2b).

### Increase in Hcy during hair growth is due to the accumulation of Hcy-keratin but not S-Hcy-keratin

The accumulation of Hcy in hair keratin (Fig. 2) can be due to *S*-Hcy-keratin, Hcy-keratin, or both. The S-linked Hcy can be liberated from *S*-Hcy-keratin by the treatment with a reducing agent such as DTT, while Hcy bound via an amide or peptide bond can be liberated from Hcy-keratin only by acid hydrolysis. To determine which form of Hcy accumulates during hair growth, we quantified *S*-Hcy-keratin and Hcy-keratin at the base of hair fibers, i.e. in young hair, and at the tip of hair fibers, i.e. in old hair, for each individual.

We found that Hcy-keratin and *S*-Hcy-keratin were present in human hair, with the levels unaffected by individual’s gender, but varied in opposite directions with the hair’s age. In young hair (0–3 months old) there was less Hcy-keratin than *S*-Hcy-keratin (82–88 vs. 178–181 pmol/mg, or 30 vs. 70%) (Table 2). In aged hair (24–60 months old) Hcy-keratin levels increased to about 500 pmol/mg, while *S*-Hcy-keratin *decreased* to about 60*–*80 pmol/mg (Table 2). Thus, in old hair there was much more Hcy-keratin (90%) and much less *S*-Hcy-keratin (10%).

**Table 2:** Hcy-keratin and *S*-Hcy-keratin levels in young and old human hair

Gender (*n*)	Hair segment age, months	Hcy-keratin (pmol/mg)	*S*-Hcy-keratin pmol/mg	Hcy/(Hcy + *S*-Hcy)	Fraction of SDS-insoluble Hcy-keratin
Female (12)	0–3	88 ± 27	178 ± 112	0.35 ± 0.14	0.62 ± 0.16
Female (12)	25–60	493 ± 491	82 ± 39	0.78 ± 0.14	0.91 ± 0.09
*P* value, old vs. young hair		0.0096	0.0135	0.0000001	0.00005
Male (7)	0–3	82 ± 41	181 ± 64	0.31 ± 0.11	0.51 ± 0.10
Male (3)	24–52	516 ± 129	62 ± 14	0.89 ± 0.02	0.98 ± 0.03
*P* value, old vs. young hair		0.00003	0.0155	0.00001	0.00005

### Hcy accumulation renders human hair keratin SDS-insoluble

Because Hcy accumulation in a protein alters or damages the protein’s structure and affects its solubility (Jakubowski 2013), we examined keratin solubility (Jakubowski 1999) at hair’s base, i.e. in young hair, and at hair’s tip, i.e. in old hair. To determine which Hcy modification might be responsible for keratin damage, we examined how the solubility of keratin is related to its *S*-Hcy-keratin and Hcy-keratin content.

**Fig. 3:**
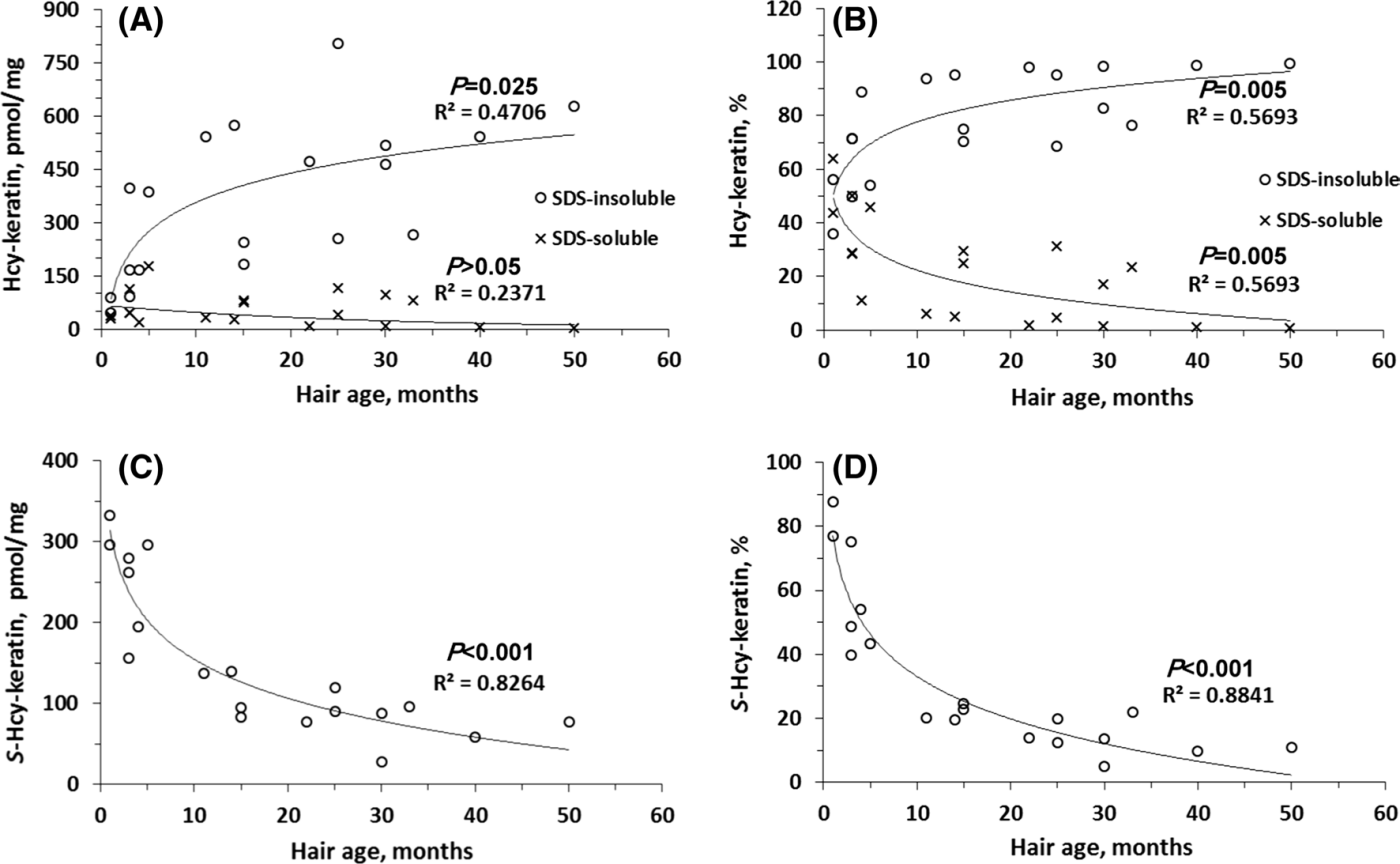
Relationships between human hair Hcy-keratin and *S*-Hcy-keratin and hair age. **a** SDS-insoluble and SDS-soluble Hcy-keratin; **b** relative values of SDS-insoluble and SDS-soluble Hcy-keratin; **c** *S*-Hcy-keratin; **d** relative values of *S*-Hcy-keratin. Hair sections (1–3 cm) of indicated age were collected from 5-year-old individuals. Each data point represents a different individual with a different hair age/length (*n* = 19, 52.6% female)

**Fig. 4:**
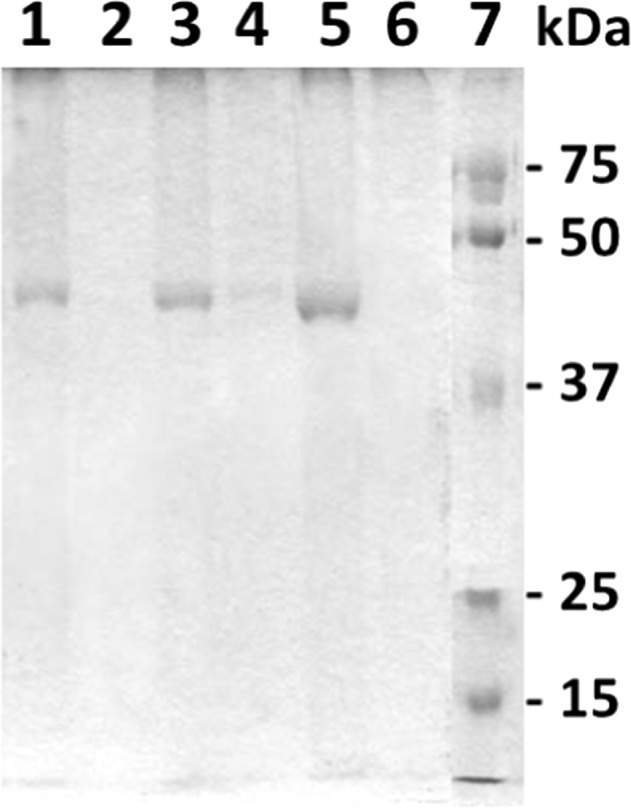
SDS-PAGE analysis of human hair keratin. Young and old hair were extracted with SDS/DTT solution as described in Materials and Methods. The extracts were analyzed on 10% SDS-PAGE gels. A keratin band is visible in samples from young hair (0–3 months) of three individuals are shown in lanes 1, 3, and 5. Soluble keratin is essentially absent in old hair (36–50 months) from the same individuals, as shown in lanes 2, 4, and 6. Lane 7 shows molecular weight standards

We found that keratin solubility was negatively correlated with Hcy-keratin and hair’s age (Fig. 3a, b). In young hair, about 50% of Hcy-keratin was SDS-soluble and 50% was SDS-insoluble (Fig. 3b). Remarkably, levels of SDS-insoluble Hcy-keratin increased (Fig. 3a), while SDS-soluble Hcy-keratin decreased (Fig. 3b) with hair’s age. In aged hair sections, essentially all Hcy-keratin became SDS-insoluble (Fig. 3a, b). SDS-PAGE analysis showed that SDS-soluble protein of 45 kDa molecular weight, corresponding to type I keratin (Langbein et al. 1999) that could be extracted from young hair sections was essentially absent in aged hair sections (Fig. 4).

*S*-Hcy-keratin in young hair (300 pmol/mg) (Fig. 3c) accounted for about 80% of total Hcy-keratin (Fig. 3d). However, there was a gradual decline in *S*-Hcy-keratin levels with the hair section age to about 50 pmol/mg, or about 10% of total Hcy-keratin at 50 months. Taken together these findings suggest that the loss of keratin solubility is caused by the accumulation of Hcy-keratin rather than the decrease of *S*-Hcy-keratin.

### Hcy-keratin accumulation is accompanied by a decrease in Met-keratin in human hair

Keratin is known to contain Met residues, which are a likely source of Hcy residues. Although hair is metabolically inactive, it is possible that keratin Met residues are demethylated to Hcy chemically. Thus, if Met-keratin is a chemical precursor of Hcy-keratin, one can predict that Met-keratin levels will be decreased in aged hair. To examine this prediction, we quantified Met-keratin levels at the tip of hair (i.e., in old hair) and at its base (i.e., in young hair) for each individual. We found that Met-keratin levels were significantly reduced in old hair compared with young hair (20.8 ± 3.3 vs. 25.1 ± 3.9 μg/mg, *P* = 0.0113), whereas Hcy-keratin levels were elevated (457.6 ± 322.7 vs. 74.9 ± 95.7 pmol/mg, *P* = 0.0012) (Table 3).

**Table 3:** Levels of Met-keratin and Hcy-keratin in young and old hair

Hair age, months	Mean ± SD	
	Met-keratin (*n* = 11), nmol/mg	Hcy-keratin (*n* = 11), pmol/mg
0–3	25.1 ± 3.9	74.9 ± 95.7
24–51	20.8 ± 3.3	457.6 ± 322.7
*P* value	0.0113	0.0012

### Hcy-keratin accumulation in human hair is positively correlated with copper and iron contents

Free Met is known to be demethylated to Hcy in the presence of cuprous copper (Cu^+^) (Lieberman and Kunishi 1965) or ferrous iron (Fe^2+^) (Baggott and Tamura 2007). This raises a possibility that the accumulation of Hcy-keratin in growing hair could be due the Cu/Fe-dependent Met → Hcy demethylation reaction.

**Fig. 5:**
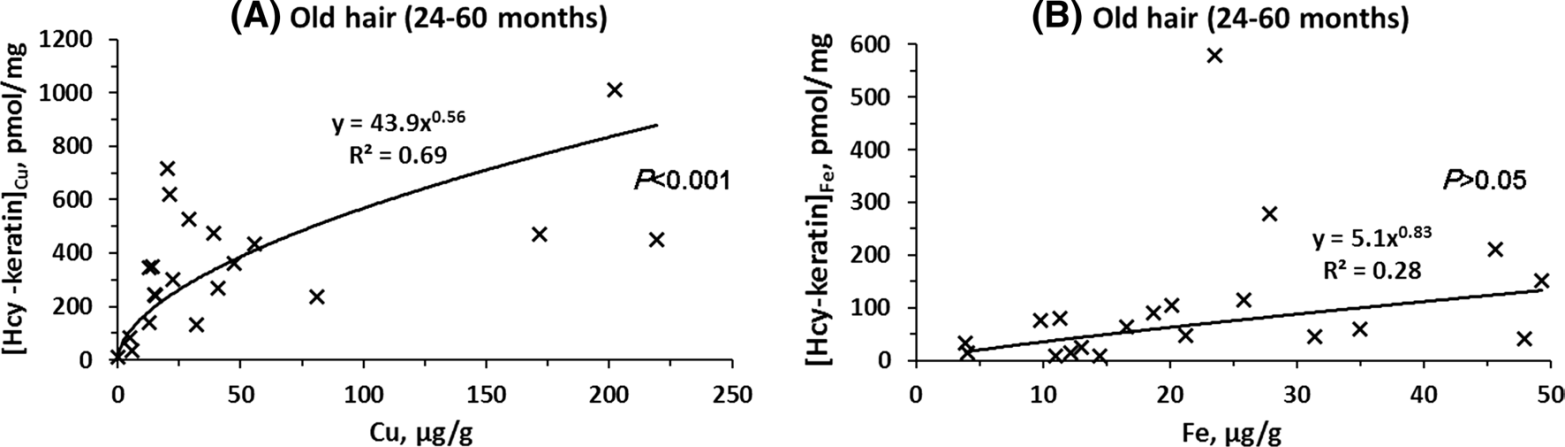
Relationships between human hair Hcy-keratin and copper (**a**) or iron (**b**) levels in 24–60-months-old hair. Contributions of copper to Hcy-keratin levels, [Hcy-keratin]_Cu_, were calculated according to a formula: [Hcy-keratin]_Cu_ = {3.5[Cu/Fe]/(3.5[Cu/Fe] + 1)}[Hcy-keratin]_Cu+Fe_, where [Hcy-keratin]_Cu+Fe_ is total Hcy-keratin. Contributions of iron to Hcy-keratin levels, [Hcy-keratin]_Fe_, were calculated according to a formula: [Hcy-keratin]_Fe_ = [Hcy-keratin]_Cu+Fe_ − [Hcy-keratin]_Cu_. The factor 3.5 is a ratio of Cu-dependent to Fe-dependent demethylation of Met to Hcy in model reactions shown in Figs. 6 and 7

To determine whether Cu or Fe can account for the accumulation of Hcy-keratin in growing hair we examined relationships between copper or iron and Hcy-keratin levels in aged hair. We found that that Cu and Fe levels were similar in the old and young hair (Table 4). We also found that there was a significant positive correlation between Hcy-keratin and copper (*P* < 0.001, Fig. 5a) but not with iron (*P* > 0.05, Fig. 5b) levels in aged hair. Copper explains 69% of the variation in Hcy-keratin, while iron might explain only 28%. These findings suggest that Cu-dependent and to a lesser extent Fe-demethylation of Met-keratin is responsible for the accumulation of Hcy-keratin in aged hair.

**Table 4:** Levels of copper and iron in young and old hair

Hair age, months	Mean ± SD	
	Copper (*n* = 14), μg/g	Iron (*n* = 14), μg/g
0–3	17.2 ± 12.4	29.9 ± 16.3
24–51	26.8 ± 22.9	30.3 ± 13.2
*P* value	0.21	0.79

### Demethylation of free methionine

**Fig. 6:**
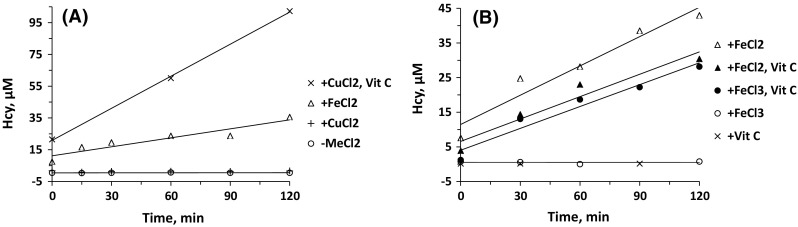
Time courses of copper- or iron-dependent demethylation of free Met to Hcy. Assays were carried out at 37 °C for 0–2 h in reaction mixtures (400 μL) containing 5 mM Met, 5 mM Na-citrate, pH 6.0, and the following additions at 5 mM each: **a** FeCl_2_ (open triangle), FeCl_2_ and vitamin C (filled triangle), FeCl_3_ (open circle), FeCl_3_ and vitamin C (filled circle), or vitamin C (times symbol); **b** CuCl_2_ (plus), CuCl_2_ and vitamin C (times symbol), FeCl_2_ (open triangle), no additions (open circle)

The relationships between Hcy-keratin and Cu or Fe in aged hair (Fig. 5) suggest that the Cu-dependent generation of Hcy-keratin is more efficient than the Fe-dependent reaction. This prompted us to examine Cu- and Fe-dependent generation of Hcy from free Met. We found that free Met was de-methylated to Hcy in reaction mixtures containing CuCl_2_ (Fig. 6a) or FeCl_3_ (Fig. 6b) in the presence, but not in the absence, of vitamin C. Because vitamin C serves as a reducing agent that converts Cu^2+^ and Fe^3+^ to catalytically active Cu^+^ and Fe^2+^, respectively, these findings are consistent with the participation of cuprous copper (Cu^+^) (Lieberman and Kunishi 1965) or ferrous iron (Fe^2+^) (Baggott and Tamura 2007) in the Met → Hcy demethylation reaction. Indeed, the Met → Hcy demethylation occurred in reaction mixtures with FeCl_2_ in the absence of vitamin C (Fig. 6b).

The rate of the Met demethylation reaction with Cu/vitamin C was 3.5-fold faster than with Fe/vitamin C. There was no Met → Hcy conversion in the absence of Cu or Fe, vitamin C, or in the presence of either CuCl_2_ (Fig. 6a) or FeCl_3_ (Fig. 6b) alone.

**Fig. 7:**
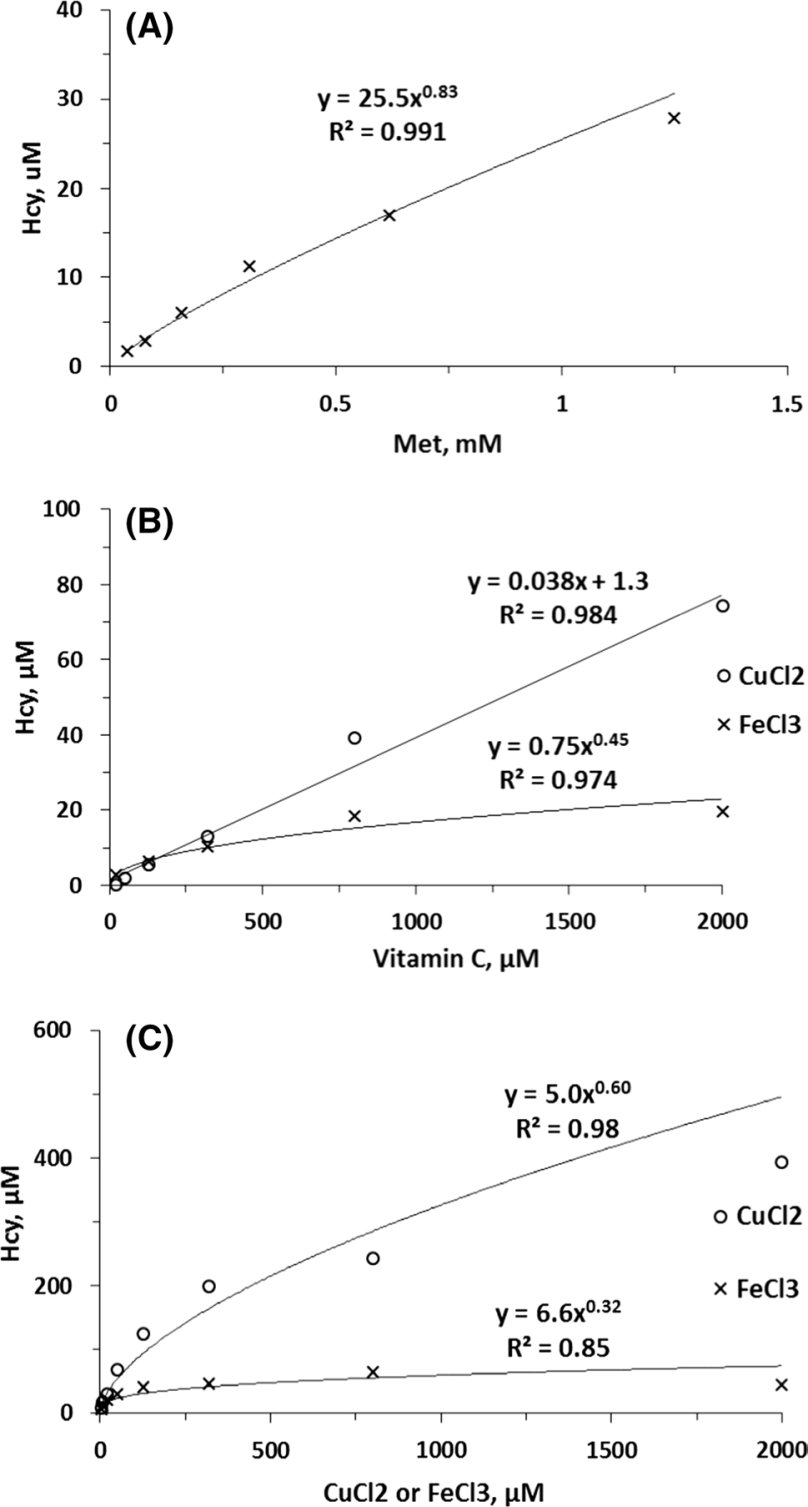
Copper- or iron-dependent demethylation of free Met to Hcy. Assays were carried out at 37 °C for 2 h in 10 mM Na-citrate buffer, pH 6.0 (400 μL) containing the following additions. **a** 0.04–1.25 mM Met and 2.5 mM FeCl_2_ (times symbol); **b** 0.02-2 mM vitamin C and 1 mM CuCl_2_ (open circle) or FeCl_3_ (times symbol). **c** 3.3–2000 μM CuCl_2_ (open circle) or FeCl_3_ (times symbol), 5 mM Met, and 5 mM vitamin C

We also found that the Met → Hcy demethylation reaction was dependent on concentrations of free Met (Fig. 7a), vitamin C (Fig. 7b), as well as CuCl_2_ and FeCl_3_ in the presence of vitamin C (Fig. 7c). The Met → Hcy demethylation reaction was up to 3.5-fold more efficient with copper/vitamin C than with iron/vitamin C over a wide range of vitamin C (Fig. 7b) and metal concentrations (Fig. 7c). Remarkably, the relationships between Hcy generation and Cu or Fe levels in these in vitro experiments recapitulate the relationship between Hcy-keratin and Cu or Fe in aged human hair (Fig. 5). Similar dependencies on Cu/Fe levels observed for Hcy-keratin in humans and for Hcy in vitro suggest that Cu/Fe-dependent demethylation of keratin Met residues is responsible for the accumulation of Hcy-keratin in growing human hair.

## Discussion

The present work shows that older sections of growing human hair accumulate Hcy-keratin with a concomitant reduction of Met-keratin levels. Our data are consistent with the mechanism of Hcy-keratin accumulation involving Cu- and Fe-dependent demethylation of Met residues in keratin. We also show that this process is associated with hair keratin damage, manifested by a loss of solubility in SDS solutions. To the best of our knowledge this is the first example of Cu/Fe-dependent protein Met → Hcy conversion associated with protein damage in humans.

Previous work has shown that proteins carry Hcy residues linked via an amide bond (Hcy-protein) (Sikora et al. 2014) or a disulfide bond (*S*-Hcy-protein) (Lim et al. 2003; Jacovina et al. 2009). Three mechanisms can account for the generation of Hcy-proteins: (1) modification of protein lysine residues by Hcy-thiolactone (Jakubowski 1999); (2) a nitric oxide (NO)-mediated mechanism affording *S*-NO-Hcy, which is a substrate for aminoacylation of tRNA^Met^ by methionyl-tRNA synthetase, forming *S*-NO-Hcy-tRNA^Met^, which in turn participates in protein biosynthesis on ribosomes by delivering *S*-NO-Hcy at positions normally occupied by Met (Jakubowski 2000a, b, 2001, 2017); (3) iron-catalyzed de-methylation of protein Met residues to Hcy (Mozziconacci et al. 2013). *S*-Hcy-proteins are generated in a red-ox mechanism involving free Hcy (Jakubowski 1999, 2000a, b; Lim et al. 2003). About three dozen of individual proteins that contain Hcy linked via an amide or a disulfide bond have been studied in vitro (Jakubowski 2013) and some of them, including albumin (Glowacki and Jakubowski 2004), fibrinogen (Sikora et al. 2014), collagen (Perla-Kajan et al. 2016), transthyretin (Lim et al. 2003), annexin A-2 (Jacovina et al. 2009), dynein (Akchiche et al. 2012), and major urinary protein (Jakubowski 2016), have been identified in vivo in humans or animals. The present study adds human hair keratin to the list of Hcy-containing proteins that have been identified in vivo.

Although in metabolically active tissues Hcy can be incorporated into proteins via amide or peptide bonds by pathways mediated by Hcy-thiolactone or *S*-NO-Hcy, respectively, both dependent on methionyl-tRNA synthetase (Jakubowski 2011, 2012, 2017), these pathways cannot be operational in metabolically inactive tissues such as hair. Thus, another mechanism must be responsible for Hcy-keratin accumulation in human hair. Our data suggest that Cu- and Fe-catalyzed demethylation of keratin Met residues to Hcy is involved. This mechanism is supported by the following evidence.

First, we show that Hcy-keratin accumulation is accompanied by a concomitant decrease in Met-keratin (Table 3); this is consistent with Met-keratin being a precursor of Hcy-keratin. Second, there are significant correlations between Hcy-keratin and Cu/Fe levels, with Cu and Fe accounting for 69 and 28%, respectively, of the inter-individual variation in Hcy-keratin in aged hair (Fig. 5). Third, the relationships between Hcy-keratin and Cu or Fe levels in human hair were recapitulated in in vitro Cu/Fe-dependent Met demethylation experiments (Fig. 7c).

Copper and iron occur in human hair (Chojnacka et al. 2005; Suliburska 2011) and the hair is constantly exposed to reducing conditions promoting demethylation of Met-keratin to Hcy-keratin. The reduced cuprous Cu^+^ and ferrous Fe^2+^cations required for the Met → Hcy demethylation reaction are most likely generated by the exposure of hair during cosmetic treatments with plant extracts or shampoos and conditioners containing vitamin C and other antioxidants (Fernandez et al. 2012). In fact, juice squeezed from cereal grasses has been shown to reduce ferric to ferrous iron (Pornprasertpol et al. 2015). Our findings that levels of *S*-Hcy-keratin, i.e., Hcy linked to keratin via a disulfide bond, were gradually decreasing with hair’s age (Fig. 3), is consistent with exposures to reducing agents such as ascorbic acid, which would reduce the disulfide-bound Hcy to free Hcy (Park 2001) which is lost during hair washing.

Previous in vitro work with model proteins (Jakubowski 2013) shows that the accumulation of *N*-Hcy-protein alters the protein’s structure/function and can lead to the generation of insoluble protein aggregates (Jakubowski 1999) with amyloid-like properties (Paoli et al. 2010). The free Hcy thiol in *N*-Hcy-protein is susceptible to one-electron redox reactions, generating intermediary radicals and/or radial ions (Schoneich 2017). These intermediates can in turn generate protein multimers bound by disulfide bonds (Jakubowski 1999; Perla-Kajan et al. 2007) and other reaction products (Sibrian-Vazquez et al. 2010). Thyil radicals produced from *N*-Hcy-protein can undergo hydrogen atom transfer generating C^α^-centered radicals, well-known precursors of protein carbonyls (Sibrian-Vazquez et al. 2010; Schoneich 2017). Indeed, *N*-Hcy-protein is susceptible to further oxidative damage (Glowacki and Jakubowski 2004), which leads to protein aggregation manifested by a loss of solubility (Jakubowski 1999). That these mechanisms are physiologically relevant is supported by findings showing that the accumulation of *N*-Hcy in motor proteins dynein and kinesin leads to aggregation of these proteins in brains of hyperhomocysteinemic rats (Akchiche et al. 2012).

The present work provides the first example of Hcy-related protein damage in humans. Specifically, we have shown that in older sections of hair human essentially all Hcy-keratin is SDS-insoluble, in contrast to the young hair sections in which only about half of Hcy-keratin is SDS-insoluble (Fig. 3a, b). Further, SDS-PAGE analysis of hair protein shows that essentially all keratin in older hair sections is SDS-insoluble and cannot be extracted (Fig. 4).

Other amino acid residues can also be damaged during protein aging (Stadtman and Levine 2003). For example, amino acids that absorb UV light, such as cysteine, tryptophan, tyrosine and phenylalanine residues in hair keratin are damaged by sun light (Robbins 2012). However, methionine does not absorb UV light and thus is resistant to sun light damage (Forbes et al. 1962). Thus, our finding that in aged hair sections essentially all Hcy-keratin is SDS-insoluble strongly suggests that Cu/Fe-dependent demethylation of Met-keratin to Hcy-keratin is responsible for hair keratin damage.

The ultimate proof for the demethylation of methionine residues in keratin would require mass spectroscopy analyses. Unfortunately, because of a complete loss of keratin’s solubility in aged sections of human hair (Fig. 4), such analyses cannot be carried out.

In conclusion, our data provide evidence for Hcy-related protein damage in humans and suggest an underlying mechanism. In this mechanism, Cu- and Fe-dependent demethylation reactions convert Met residues in keratin to Hcy, forming SDS-insoluble Hcy-keratin in human hair. Although additional experiments are required to verify whether this mechanism is widespread and affects other proteins, our results provide a new understanding of protein damage.

## Abbreviations

DTT: Dithiothreitol
Hcy: Homocysteine
HPLC: High performance liquid chromatography
Hcy-protein: Hcy linked to a protein via an amide (peptide or isopeptide) bond
*N*-Hcy-protein: Hcy linked to a protein via an isopeptide bond
*S*-Hcy-protein: Hcy linked to a protein via a disulfide bond
Met: Methionine
NO: Nitric oxide
SDS: Sodium dodecyl sulfate
